# Mobile Phone-based Intervention to promote un-interrupted HIV treatment during the COVID-19 pandemic

**DOI:** 10.4314/ahs.v22i2.14S

**Published:** 2022-08

**Authors:** Damalie Nakanjako, Eisah Kakyama Mayanja, Agnes Semwanga Rwashana, Fred Semitala, Cordelia Katureebe, Mina Ssali, Martin Muddu, Isaac Ssinabulya

**Affiliations:** 1 Department of Medicine, School of Medicine, Makerere University College of Health Sciences, P.O. Box 7072, Kampala, Uganda; 2 New Wave Technologies, P.0.Box 24159, Kampala, Uganda; 3 Department of Information Systems, College of Computing and Information Sciences, Makerere University, P.O. Box 7062, Kampala, Uganda; 4 Makerere Joint AIDS Program, Kampala, Uganda; 5 Ministry of Health, HIV treatment program, Kampala, Uganda; 6 Uganda Heart Institute, Kampala, Uganda

**Keywords:** M-Health, patient follow up, Chronic HIV treatment, Antiretroviral therapy

## Abstract

**Introduction:**

Keeping HIV-infected adults away from the health care system during the COVID-19 travel restrictions, presents a challenge to HIV treatment adherence.

**Methods:**

This study focused on the initial two phases where Phase 1 designed a Makerere College of Health Sciences (MakCHS) Unstructured Supplementary Service Data (USSD)-based application; and Phase 2 piloted patient enrolment onto the application and determined the feasibility of remote follow-up of patients receiving long-term antiretroviral therapy (ART).

**Results:**

A off/online user application, MakCHS Health app, was developed. Overall, 112 patients [(66(59%) female] receiving ART at Mulago ISS clinic, Kampala, were enrolled onto the MakCHS Health app. Up to 89 (80%) utilized the app to access medical help. Patients' medical queries included needs for drug refills, missed taking HIV medication, medical illnesses, access to COVID-19 vaccination and other personal needs that required clinicians' attention.

**Conclusion:**

Piloting a MakCHS Health application for patient follow-up was feasible and well-received by HIV treatment providers and patients receiving ART. We recommend scale up of the application to enroll all patients receiving long-term treatment for HIV/AIDS, and subsequently expand to. other HIV treatment programs in similar settings.

## Introduction

In Uganda 1.6M people are living with HIV and 23,000 died of AIDS-related illnesses in 2018. Only 73% of HIV-positive adults are receiving antiretroviral therapy (ART), and only 64% of all people living with HIV have suppressed viral loads^1^. HIV-associated dysfunction of both innate and acquired immunity may not recover completely despite long-term antiretroviral therapy^2^–^6^. Hence the need for uninterrupted HIV treatment and adherence monitoring to promote sustained viral suppression and management of relevant co-morbidities among ART-treated individuals. To limit the spread of the COVID-19 pandemic Uganda instituted a protracted lockdown that banned public/private transport, and sick individuals required a ‘special letter’ from the district commissioner to travel to hospital. These measures likely affected HIV-infected individuals, particularly given the stigma associated with the disease. Consequently, keeping HIV-infected adults away from the health care system, as happened inevitably during the COVID-19 lockdown and various travel restrictions, presents a challenge to HIV treatment adherence due to delayed or lack of drug refills or missing medication due to acute illness, drug-related toxicities, and/or psychosocial factors associated with the COVID-19 pandemic such as anxiety disorders, domestic violence and stigma. Moreover, there were limited options for virtual patient medical care for people living with HIV at the time. Without interventions to support continuity of HIV treatment, patients receiving ART are likely to enter a new crisis of AIDS-associated morbidity and mortality due to treatment failure and opportunistic infections among patients that have had poor adherence to medication during the COVID-19 crisis^7^. To fill this gap, we designed and piloted a Mobile Phone-based patient follow-up package with virtual Medical Interventions (PMI) for patients receiving HIV treatment at the Mulago Immune Suppression (ISS) clinic under the Makerere Joint AIDS program to support adherence to medication and self-care^8^.

PMI consists of

- a Mobile phone app platform that allows real-time interaction of patients and care takers with the HIV treatment provider to handle patient queries that may hinder their adherence to medication. The mobile app flags the multi-disciplinary team to provide customized support to the patients' needs. For patients that need to see a doctor, the app was programed to link them to the ART provider (Nurse/counselor/doctor/psychologist) to respond to the patients' needs on phone depending on the triaging information provided. Where drug refills were requested, patients were linked to a drug delivery system including delivery with a motorcycle. Physical appointments were reserved for patients who needed to see a medical doctor (and where symptoms persist despite the virtual medical interventions).
- A dedicated ART nurse-counselor to follow up the patients and their caretakers weekly,
- available support from a Clinical Psychologist/Psychiatrist to provide virtual individual and family psychosocial support in regards to adherence to HIV medication as required by the patient, and
- A dedicated clinician to review co-morbidities and any drug-related side effects and drug-drug interactions.

This paper describes the process of designing and set up of the MAKCHS portal to follow up patients receiving ART and the pilot for the app by the first clinicians and patients to enroll.

## Methods

### Study design

This proof-of-concept study was implemented in two phases.

**Phase 1:** Designing the Mobile Phone-based patient follow-up Package with virtual Medical Interventions. The project was presented to the Ministry of Health HIV care team and the M-health team who both welcomed the idea. The implementation was done through a collaborative agreement with National Information Technology Authority-Uganda (NITA-U) to open an M-health portal for Makerere University College of Health (MakCHS Health Clinic) to receive various SMS traffic to and from patients enrolled on the program.

Questions were piloted according to the essential information required to monitor patients receiving HIV treatment adherence, drug stock and any clinical events that may affect the patient's ability to take medication. Time out due to inactivity was adjusted to allow patients enough time to read and respond to prompts on the phone (that are similar to prompts used for phone-based payment platforms. The Unstructured Supplementary Service Data (USSD)-based interactions were accessed by both individuals with and without smart phones. The response pathway for clinicians was also developed. Clinicians (doctors and nurses) and peer counselors were trained on how to enroll patients on to the app, how to respond to patients' queries and how to link to the drug delivery system to home care teams. The principal investigator collaborated with Medly Uganda, who were also co-investigators on this project. The team previously worked together on developing a successful project on m-Health interventions for patients with heart failure.

**Medly Uganda System Overview:** Medly Uganda provided technical expertise on providing a user-centred design process for low-cost feature phones using a simple interface with an algorithm that generates self-care instructions based on patient-reported symptoms alone. The app was based on the USSD platform, a universally available interface that enables users to navigate hierarchical menus and offers multiple advantages for mHealth including enhanced security features, verifiable transactions, simple navigation, and real-time interaction. It was developed using RapidPro, an open-source platform that allows simple creation and deployment of mHealth systems. Within the Medly Uganda system, we designed a simple, secure, web-based dashboard for clinicians to monitor safety alerts generated by the app based on patient symptom reporting. Using these platforms, we embedded the Medly Uganda system onto a subcode on NITA-U, a government-endorsed, hybrid USSD-SMS platform with proven feasibility and user engagement.

**Figure 1:**
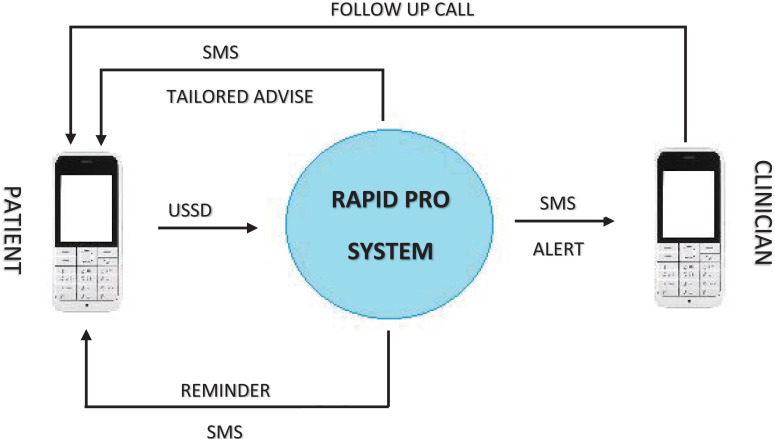
Illustration of the patient-clinician Unstructured Supplementary Service Data (USSD) - SMS pathway

**Figure 2:**
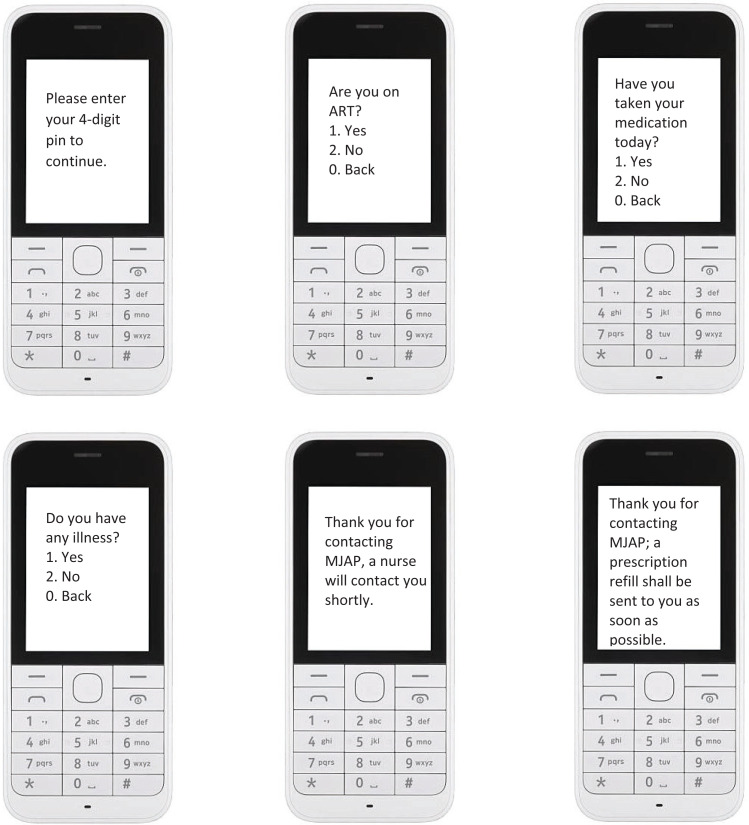
Illustrating the patient interface with the USSD interactions with questions adherence to medication and any current illness

**Unstructured Supplementary Service Data (USSD) Overview:** USSD is a signaling protocol usable on any GSM-enabled mobile device, whether the device is a smartphone or a feature phone (i.e. non-smartphone). An interaction with the system is initiated by the user dialing a USSD code on their mobile phone, which connects their device a computer on the service provider side. This connection allows the user to navigate through a decision-tree menu within a microbrowser on their phone. Though the channel makes use of a microbrowser, one major benefit of USSD is that it does not require internet connectivity or airtime on the user's device, but solely a connection to a mobile network (Figure 1). We leveraged experience from the previously developed mHealth Heart failure application by the Medly Uganda team and made adaptations to suit the necessary HIV remote care support^9^,^10^. Given the USSD and SMS aspects of the Medly Uganda system, the RapidPro system (Figure 2) was utilized to facilitate two major components. First, system uses a USSD flow involving the designed decision-tree algorithm to determine the patient's status on a given day. Completion of the USSD flow triggers the start of an SMS flow, where the system uses the determined patient status to send the appropriate SMS messages to the patient and clinician (if required). Patients' responses were based on symptoms to aid the clinician make differential diagnoses and make calls for any additional information, and invitations to the clinic if a physical assessment was deemed necessary.

**Patient-Facing System:** To initiate the system, patients are required to dial into MakCHS using a USSD code on their mobile device. Once engaged with the USSD system, the patient responds to a series of yes-or-no questions pertaining to their HIV treatment (Figure 2). Upon receiving the patient's symptom data, the system autogenerates self-care advice that is sent to the patient via an SMS message. For patients that report severe symptoms, the system also sends an SMS to a reporting clinician, containing a brief summary of the patient's symptoms and a number at which the patient can be reached at any time of the day, to respond to the patients' needs. The nurse or doctor reviews the patient's complete history in the linked clinic database (either on their computer or smartphone), before following up with the patient.

**Data Storage and Movement:** All patient responses to the symptom questionnaire are stored within their ISS clinic facility. Aside from the symptom data, the only other patient information that is stored on the RapidPro server is the patient's phone number, their preferred language and a four-digit PIN that they use to access the system. The remainder of the patient-specific data is stored separately on the ISS clinic -hosted server, along with clinician login credentials and clinician-recorded notes. The dashboard would then use patient phone numbers, which is stored on both the ISS clinic and RapidPro servers, to query data from both databases and to present it to the clinician in an efficient manner.

The patient interface had questions about whether the patient was taking medication, reasons for missing medication (for those who had missed their medication) and any complaints or illness (Figure 2). These responses were received on the clinician's side as sms alerts for medical attention and the HIV care provider (nurse, counselor, doctor, psychologist/Psychiatrist) responded as required, depending on the patients' situation. A clinicians' algorithm for response was embedded, based on the patients' responses and clinicians were able to respond appropriately and sent daily advise based on SMS and/or phone call assessment of the symptoms. All patient interactions were linked to the clinic database to allow clinicians to make informed decisions after reviewing all patients' clinical information and to allow continuity of the phone-based interventions with the clinic-based interventions. Each week, a specific clinician was scheduled to respond to patients' responses.

**Phase II: Piloting the PMI among patients receiving HIV treatment at ISS clinic**: This was a prospective proof-of-concept study among patients receiving ART at Mulago ISS clinic.

### Study population and sample size

The Mulago ISS clinic offers HIV care to approximately 17,000 individuals under the Makerere Joint AIDS program (MJAP) and 200–260 attend the clinic daily. For the proof-of-concept study, we signed up a random number of 112 patients who had received HIV treatment for at least six months, lived within a 20km radius around Kampala city centre and provided informed consent to participate in the study.

**Patient selection:** Adult patients were randomly selected from the patient database of adults receiving ART at the Mulago ISS clinic for at least six months and residing within a 20km radius around Kampala City Centre. We excluded patients who did not have any contacts (phone number or physical address of his/her caretaker/next of kin) in the clinic database.

### Study procedures

We offered medical follow up on phone and reserved physical appointments for those patients who required to see a doctor for specific conditions, and where symptoms persisted after the virtual medical intervention. Upon provision of written informed consent, obtained by a study nurse/counselor, patients were enrolled onto the mobile Health HIV application and taken through the procedures of using the mobile app. To determine the feasiblity, and factors that might affect the acceptability of using the mobile phone-based patient follow up package by clinicians and patients, we conducted several meetings with clinicians and expert patients (peer counsellors) throughout the development and implementation processes of the project. The study team performed various iterations with providers and People Living with HIV (PLHIV) at the ISS clinic to refine the application. We then identified expert peers from PLHIV and on-boarded them onto the system to test flows, improve on wording and look out for time out with the system; after which we enrolled 112 participants and four nurse counsellors to support the proof-of-concept phase.

### Data management and confidentiality

Project data was collected and managed using our Medly Uganda app database that is hosted by the MOH mhealth platform from National Information Technology Authority-Uganda (NITA-U) and was stored on the clinic servers at Makerere Joint AIDS Program (MJAP). Patients' data was password protected and each patient had an individual password protected account linked to their unique phone and mobile money account. Locally, at the clinic, project data was stored securely in a password protected computer and server, only accessible to the study team using electronic signatures, password aging, and lockout rules. This study was monitored by the MJAP research regulation team.

### Data analysis

The process of developing the intervention (Phase 1) was documented. Lessons learned were documented and discussed with the various stakeholders right from the design through implementation of the project. Data of patients enrolled in the app (Phase 2) was analyzed and presented using figures and frequency tables. Feasibility of the proof-of concept was determined by the output which was the application itself and the ability to complete sms patient-to-doctor interactions that yielded a medical intervention. Acceptance of using the app by clients was determined by the proportion of the patients approached who accepted to be enrolled onto the program. Observations were described from patients who used the app at least once to request for any form of medical support from the clinic.

## Ethical considerations

The record of consent was stored at the ISS clinic under lock and key, with access limited to the study team. To safeguard against patients' complaints about third party use of their phones, patients' accounts on the app were password protected and linked to the respective clinic identification number. Study staff explained, in the informed consent form, that all participants were still welcome to the clinic if they felt that the virtual support was not enough to meet their needs at the time of interaction with the system.

The psychosocial team, consisting of a counselor, clinical psychologist and psychiatrist, were available to address any psychosocial issues among patients and staff during the study period. Any other social issues or conflict were handled by the study team leadership and the human resource to ensure smooth implementation of the project. This study was approved by the School of Medicine Research and Ethics Committee and the National Council for Science and Technology.

## Results

MAKCHS health app was successfully developed in collaboration with NITA-U, Medly Uganda, RapidPro and New Wave Technologies, including the minimum set of questions required for virtual assessment of patients receiving ART. The app was deployed and both patients and health care providers were enrolled as summarized in figures 1–2. Iterative involvement of patients and clinicians in the app development process helped to develop precise questions that patients could respond to promptly, as well as an in-build algorithm to provide alerts to the clinician based on the respective patients' entries.

Overall, 112 patients receiving ART at the ISS clinic were enrolled on to the MakCHS health app for this proof-of-concept period; of whom 66/112 (59%) were female. The mean age of 44.9 years; and patients had received ART for a mean duration of 9.2 years. Up to 89 individuals (80%) utilized the app to access medical help from the ISS clinic (Table 1). Patients' medical queries included needs for drug refills, complaints of illnesses that hindered them from taking their HIV treatment, how to access COVID-19 vaccination and other personal needs that required attention of a clinician (Table 2).

**Table 1:** Summary data of 112 patients receiving antiretroviral therapy who were onboarded onto MakHealth app during the proof-of-concept period

Female gender	66 (59%)
Mean age in years	44.9
Mean duration of ART in years	9.2
Language used in MakHealth app	
Luganda	87 (78%)
English	25 (22%)
Utilization of MakHealth app	
At least once	89 (80%)
Not yet utilized	23 (20%)

**Table 2:** Sample information received from patients who sought medical attention through the MakHealth application for patient follow up and the actions triggered from the clinic

Information/queries received from patients	Action triggered from the clinic
**From patients not taking medication**	
Just forgot 3 (23%)	Counselor called for adherence support
Medical illness 3 (23%)	Clinician called and advised
No medication 4 (31%)	Medication was packed and delivered
Other trouble 3 (23%)	Clinician called the patients and handled accordingly

**Patients who need drug refills within 2–3** **weeks**	Medication was packed and delivered

**Patients who asked about COVID-19** **vaccination**	Appropriate information was given Patients were directed to access COVID-19 vaccine at a location nearest to them

## Lessons learned

We established a collaborative framework with memoranda of understanding that were signed and executed by the different partners; NITA-U, MakCHS, RapidPro, Medly Uganda and New Wave technologies who were key players in this proof-of-concept project to demonstrate the feasibility of using the USSD-based app to transmit and utilize information for virtual medical interventions. This project demonstrated the feasibility of leveraging already existing USSD platform to develop a mobile application to benefit the vulnerable population of HIV-infected adults receiving HIV treatment, and unable to access the health care system due to limited movements and social distancing guidelines to prevent community spread of the COVID-19 pandemic. Collaborating with existing platforms including Medley Uganda, RapidPro, NITA-U and New wave technologies, among others helped to reduce the development period by not “re-inventing the wheel”.

Both face-to-face and virtual meetings with clinicians, patients and their caretakers, and the Ministry of Health HIV treatment group were useful through implementation of the project during the COVID-19 pandemic to overcome the COVID-19 related travel restrictions and social distancing protocols. Continuous iterative dialogue between the app developers, clinicians and patients was relevant to inform relevant modification of project activities in line with the dynamic community-level social distancing plans and national COVID-19 prevention guidelines. All the patients were able to interact in Ganda (80%) and English. However, scaling up the coverage to patients outside Kampala may require additional languages. We noted the need for a dedicated team of clinicians and IT personnel to respond to patients' queries in real time. Patients preferred responses by phone calls to sms responses from clinicians. The calls gave patients more time to interact and receive specific responses from the clinicians and counselors.

Whereas peer counselors helped to ensure smooth enrollment of patients on the app at the clinic, enrolling patients off-site needs to be further developed, particularly for patients who did not have smart phones.

The mobile phone-based initiative to follow up patients receiving HIV treatment was well received by health care providers and patients who participated in the pilot. We recommend scale of the enrollment to include all patients in care at the ISS clinic and subsequently all people living with HIV in Uganda and similar LMICs.

## Limitations

This pilot study was limited to patients who had received HIV treatment for at least six months and may not be generalizable to patients initiating ART. More research is needed to pilot this approach among newly-diagnosed patients initiating ART who may need more frequent interaction with clinicians. Another limitation is the fact that qualitative data was not collected from the patients enrolled on the app. However, we pre-tested the app among PLHIV as expert patients to inform the app development process (phase 1) and subsequently the proof-of-concept was well received by the target group of patients with at least six months of ART. This study proves the concept that patients receiving ART for chronic HIV can be monitored virtually and given relevant medical interventions that do not require physical clinic visits. We recommend collection of qualitative data to inform the scale up phase of enrolling more patients on the app as well as more translations to other dialects used in other parts of the country. Similarly, there is need for scale up studies to evaluate the const-effectiveness of the intervention among all the 17,000 patients in the ISS clinic, and subsequently to other HIV treatment programs. This study did not make provisions for patients who are unable to read and select any options provided in their local languages including the illiterate and the blind; and we propose to address this in the next phase by including voice capture and artificial intelligence technologies.

## Conclusion

MakCHS health app for virtual follow up patients was successfully developed and we proved the concept that ART-treated individuals and HIV care providers are able to use the app for relevant virtual medical interventions to maintain continuity of care. We recommend scale up of the application by enrolling all patients receiving long-term treatment for HIV/AIDS at ISS clinic and subsequently expand to. other HIV treatment clinics in similar settings.
